# Beyond correlates: the social gradient in childhood overweight

**DOI:** 10.1186/s13690-023-01232-x

**Published:** 2024-01-09

**Authors:** Lina Hermeling, Jürgen M. Steinacker, Susanne Kobel

**Affiliations:** Division of Sports- and Rehabilitation Medicine, Centre of Medicine, Ulm University Hospital, Ulm, Germany

**Keywords:** Obesity, Preschool children, Parents, Misclassification, Weight status

## Abstract

**Background:**

Health (in)equity has a high priority on research and policy agendas. Even though it is known that inequalities in overweight prevalence accumulate with age and are already existent among children below the age of six, research on this topic is scarce. In this young age group, parents play an important role in preventing overweight and associated adverse consequences. This study examines the magnitude of parental misclassification of child weight status and its correlates, focussing on the factors that determine social status and equity.

**Methods:**

Preschool children’s weight and height was measured objectively. Parents gave information on their socioeconomic background. Family education was dichotomised into tertiary and non-tertiary educational level, according to CASMIN. Binary logistic regression, adjusted for parental BMI, was applied to detect odds of childhood overweight.

**Results:**

Data on family educational level and anthropometrics were available from 643 children (4.5 ± 0.82 years, 52.7% male) and their parents of which 46.5% (n = 299) had a tertiary educational background. The groups (tertiary vs. non-tertiary educational level) differ significantly in overweight prevalence (3.7% vs. 11.9%, p ≤ 0.001). Odds of overweight were two times higher in children with non-tertiary educational background (OR: 2.123, CI: 1.010–4.461, p < 0.05), adjusted for parental BMI.

**Conclusion:**

Children from families with low educational background have an elevated risk of overweight, already at a very young age. Education in general (not explicitly health education) seems to play a tremendous role in the prevention of overweight and obesity and should therefore be implied in policies enhancing health equity.

**Trial registration:**

DRKS-ID: DRKS00010089.

**Table:** 

Text box 1. Contributions to the literature
• Health-related risks occur more frequently in families, especially in those from a low educational background. Children from such families have an elevated risk of being overweight even at the age of 3 to 5 years.
• Children are also more likely to be overweight if their mother is overweight, if the child grows up in a family with a migration background, and a low household income.
• Parental correct classification of their child’s weight status is essential to childhood health. If the child and/or the parent is overweight, the likelihood for a parental misclassification of their child’s weight status is most likely.

## Background

### Childhood health differences

Health equity has a high priority on research and policy agendas worldwide. Nonetheless, gaps in health and well-being are extremely persistent while challenges are constantly changing. Global inequities, armed conflict and violence, globalisation, nuclear proliferation, forced migration, and climate change are global child health issues that violate children’s rights to optimal health and development [1]. These issues affect children’s physical and mental development with lasting consequences on several levels.

Health disparities can be found on global, national and regional level. The European Health Equity Status Report [2] states manifested gaps in self-reported health for boys and girls. These inequities in health and well-being last into adulthood despite the offering of universal and accessible primary-level education in almost all countries in the World Health Organisation’s (WHO) European Region [2].

The scientific field of syndemics, as a conceptual framework for understanding health conditions which examines why certain health conditions cluster, encourages a deeper understanding of the interplay of social and environmental factors on global health and wellbeing, not only recognizing comorbidities and covariates but seeing the underlying mechanisms and dynamics. Research shows that health-related risks occur more frequently in families [3]; for example, body weight [4], cardiovascular risks [5], and body fat distribution [6] clusters in families. Such incidents can be explained by both genetics and factors of shared (common) family life, such as the family’s socio-demographic situation (e.g. socio-economic status, level of education) as well as family status and structure (e.g. children’s age, patch-work family, single parent, etc.), which are associated with health outcomes [7]. Syndemics is therefore especially useful in the context of social inequality [8], which is why this biopsychosocial approach will be applied here.

The socioeconomic health gradient is seen in several health indicators already in early childhood. These are for example mortality but also preventive care such as immunization and chronic illness management [9], which often depend on a wide range of variables including fetal-maternal physiology, maternal (paternal, family) mental health, infant nutrition, and the physical and emotional home environment, including socio-economic factors [10]. In scope of this study, childhood weight status is examined as an indicator that affects according to the World Health Organisation almost a third of children in the European Region [11]. 31% of boys and 28% of girls aged 7–9 years suffer from overweight [11] but at the same time, it can be observed, that both, girls and boys, in more affluent households are more likely to be physically active, according to the OECD PISA index [12]. A number of studies have reported socioeconomic inequalities in children’s and adolescent’s health behaviours and weight status in high-income countries, with children and adolescents from lower socioeconomic status households typically having higher Body-Mass-Index and an increased risk of obesity [13, 14].

Even though it is long known that inequalities in overweight prevalence accumulate with age and are already existent in early childhood, data and research on this topic are scarce, especially among children below the age of six. Health- and weight-related inequalities vary on the basis of socioeconomic status and cultural background. It is well established that children from families with a low socioeconomic status are at higher risk for overweight and obesity as dietary and physical activity habits are formed by sociocultural and societal factors [15, 16].

Overweight in childhood can track into adolescence and adulthood, implying substantially elevated risks of non-communicable diseases, diabetes type 2 and psychosocial constraints [17]. Further, overweight children suffer from delayed skill attainment already in kindergarten [18] and tend to achieve lower grades in school [19, 20]. Beyond that, they may experience social exclusion and stigmatisation [21].

Especially in this young age group, parents play an important role in the prevention of overweight and associated adverse consequences through regulating diet and leisure time activities, but also through providing beliefs and showcase behaviour [22, 23].

### Parental perception of children’s weight status

Parental perception of their child’s weight status is a key factor in the prevention and treatment of overweight and obesity but it tends to be underestimated in preventive interventions. Parents of overweight children tend to underestimate the weight status of their children [24–26]. A meta-analysis of 78 studies (n = 15 791) reveals that 50.7% of parents underestimate their overweight or obese children’s weight, while 14.3% underestimate their children’s normal weight status [24]. Also, highly-educated obese parents are more likely to provide negative misclassification of their child’s weight status, which, however, may be caused by a social desirability bias [27].

Yet, the magnitude and direction of childhood weight status misperception changes with age. In the youngest age group, the proportion of overweight children was overestimated, while it was underestimated for older children and adolescents [28].

To date, only few studies have robust multivariate analyses, which include a variety of socio-economic factors and their interactions. Therefore, the present study provides an in-depth analysis of moderators of parental misperception.

## Aims

The aim of this study was to analyse inequalities in childhood overweight prevalence and perception and to examine possible associations with cultural, economic and educational background. In addition, this research examines the magnitude of parental misclassification of child weight status and its correlates, focussing on the influence of factors that determine social status and equity.

## Methods

This cross-sectional analysis used baseline data from the cluster-randomised *Health Survey*, which is an evaluation study within the health promotion programme “Join the Healthy Boat”, a multicomponent setting-based programme that aims at a healthy lifestyle of kindergarten children and supports among others the prevention of overweight and obese children. For that, kindergartens were recruited in southwest Germany; children were eligible to take part if they were between three and five years old at the time of baseline measurements and their parents provided signed consent. Further details on the study design, recruitment and evaluations of the H*ealth Survey* can been found elsewhere [29]. The study is registered at the German Clinical Trials Register, German Institute of Medical Documentation and Information, Cologne, Germany [DRKS-ID: DRKS00010089] and the study protocol was approved by the ethics committee of the local University (Application Number 188/15) as well as the German Ministry of Culture and Education.

### Participants

In total, 973 children, between three and six years of age, visiting 57 kindergartens in Baden-Württemberg, southwest Germany, were examined. Since only children with a complete dataset were included, during data processing, 123 children were excluded due to missing information (e.g. height, weight or age). Accordingly, 850 preschool children (4.56 ± 0.83 years, 51.4% male) and their parents were included in this statistical analysis for secondary outcomes; primary analyses of the evaluation study have been published elsewhere [30].

### Data collection

Children’s weight status includes Body-Mass-Index (BMI), BMI percentiles and Waist-to-Height-Ratio (WHtR). Examinations were performed according to the standards of the International Society for the Advancement of Kinanthropometry (ISAK) by trained examiners in small groups of three to four children, separated by gender [31]. Measurement of body weight was performed using calibrated flat scales (model 826, Seca® Company, Germany) in minimal clothing. Height was measured barefoot with mobile stadiometers (model 217, Seca® Company, Germany). Waist circumference was measured halfway between the lower costal border and the iliac crest using a metal tape measure (Lufkin® model W606PM, Lufkin Industries Inc., Texas, USA). Children’s BMI was calculated as weight divided by height squared (kg/m²). BMI percentiles (BMIPCT) were allocated based on age- and gender-specific charts of international cut-off criteria, defined to pass through body mass index of 25 and 30 kg/m^2^ at age 18 (overweight and obesity, respectively), in order to classify children into weight categories underweight, normal weight and overweight/obese [32]. In addition, WHtR as a measure of central obesity (WHtR > 0.5) was calculated as the ratio of waist circumference to height in centimetres [33].

Levels of academic and professional education as well as monthly net income from both parents were assessed within a parental questionnaire, which incorporated validated questions from a questionnaire repeatedly used in a national sample with more than 30,000 children [34]. Family level of education was categorised according to the adjusted “Comparative Analyses of Social Mobility in Industrial Nations” (CASMIN) classification [35]. Levels were dichotomised into tertiary and elementary/intermediate level of education. Household monthly net income was assessed on a seven-point-scale and dichotomised into < 1750€ and ≥ 1750€, according to Winkler & Stolzenberg [36]. Additionally, children were classified as having a migration background if they were born abroad or at least one parent was born abroad. Children not having a migration background were titled “native”. Parental weight status was assessed subjectively by asking mother and father separately for their height (in cm) and body weight (in kg), which was subsequently calculated into their Body-Mass-Index (BMI) by dividing weight by height squared (kg/m²). To classify parental weight status (mothers and fathers separately) cut-off points recommended by WHO [37] were used to determine normal weight (including underweight; BMI ≤ 24.9) and overweight (including obesity; BMI > 25).

Parental perception of their child’s weight status was assessed for mothers and fathers with the following question:

*DoDo you think your child is: (1) very underweight, (2) underweight, (3) normal weight, (4) overweight, (5) very overweight?*

This is the key variable used to identify misclassifications in comparison to the objectively measured body composition within the sample.

In order to assess the extent of parental misclassification, children’s objectively assessed weight status (using the values 1–5 for very underweight until very overweight, determined by international cut-off points [32]) was deducted of subjectively assessed parental classification of their child’s weight status (also using values 1–5, as shown when describing the asked question).

### Analysis

Data analysis was performed using IMB SPSS Statistics 25 (SPSS Inc., Chicago, IL, US). Significance level was set to α < 0.05. Socio-demographic characteristics of the sample were described. Overweight and obesity prevalence, according to international cut-off points [32], WHtR and socioeconomic factors were reported. Pearson’s Chi²-Test and Mann-Whitney-U-Test were used to reveal group-differences. Cohens Kappa (ĸ)/weighted kappa (κ_w_), a measure of interrater-reliability, was used to analyse misperceptions/agreement between children’s objectively measured weight status and parent’s perception of it. Cross-classified tables were used to assess multicollinearity. Binary logistic regression was employed to analyse strength of associations between the degree of misclassification, socioeconomic status, cultural background and parental weight status. Regressions were run separately for each explanatory variable and block wise with forced entry, adjusted for age, gender and parental BMI. Results were presented as odds ratios (OR) with 95% confidence intervals (CI).

## Results

Descriptive characteristics of the sample can be found in Table 1.

**Table 1:** Descriptive characteristics of the study population; Health study, Germany

	Missing values	Total (N = 850)
Age, years [m (SD)] *		4.56 (0.83)
Gender, male n (%)		437 (51.4)
BMI, kg/m² [m (SD)]		15.57 (1.43)
BMIPCT [m (SD)]		50.43 (26.40)
Overweight (incl. obesity) IOTF, n (%)		84 (9.9)
Obesity IOTF, n (%)		16 (1.9)
Paternal BMI, kg/m² [m (SD)]	272	26.38 (4.48)
Maternal BMI, kg/m² [m (SD)]	233	24.36 (4.66)
Migration background, n (%)	212	199 (31.2)
Family net income < 1 750€, n (%)	245	56 (9.3)
Family education medium/low, n (%)	207	344 (53.5)
Single parent, n (%)	185	49 (7.4)

*m (SD)* mean (standard deviation), *BMI* body mass index, *BMIPCT* BMI percentiles, *IOTF* Age and gender-specific cut-off points defined by International Obesity Task Force to pass through body mass index of 25 and 30 kg/m^2^ at age 18 [32]

Data on family educational level and anthropometrics were available from 850 children (4.56 ± 0.83 years, 51.4% male) and their parents of which 46.5% (n = 299) had a tertiary educational background. The groups (tertiary vs. non-tertiary educational level) differ significantly in overweight prevalence (11.9% vs. 3.7%, p ≤ 0.001).

As shown in Table 2, children were more likely to be overweight if their parents had an intermediate/low educational background, if their mother was overweight, if the child grew up in a family with a migration background, and a low household income). Parents were also more likely to be overweight when their educational background was low (paternal: OR: 0.522, CI: 0.380–0.718, p < 0.001; maternal: OR: 0.645, CI: 0.431–0.964, p < 0.05).

**Table 2:** Binary logistic regression of child weight status; Health study, Germany

Predictors	Total (n = 850)		
	OR	95% CI	*p*
Intermediate/low educational background*	2.123	1.010; 4.461	< 0.05
Maternal overweight	0.053	0.015; 0.091	< 0.05
Migration background	0.561	0.385; 0.818	< 0.005
Low household income	0.017	0.031; 0.003	< 0.05

*OR* odds ratio, 95% CI 95% confidential interval; *) adjusted for parental BMI

**Fig. 1:**
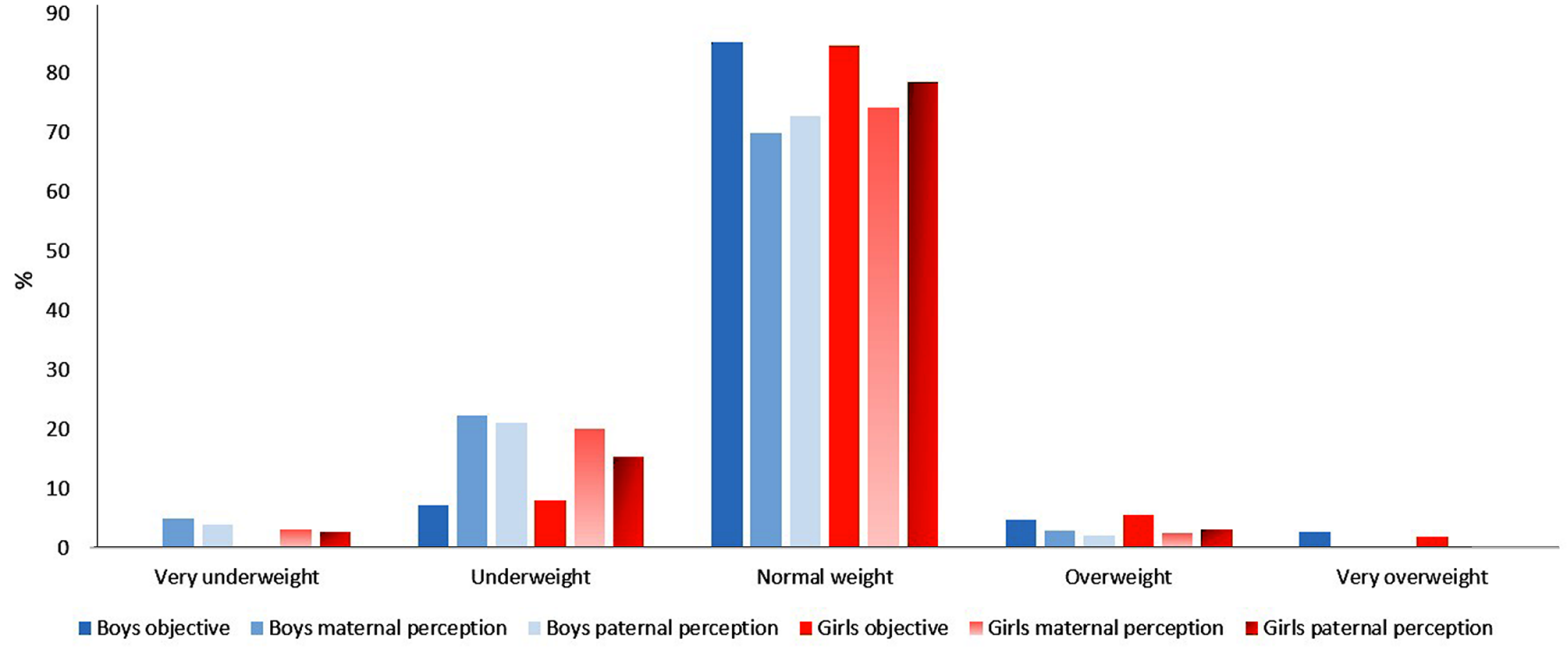
Distribution of children’s weight categorisation for girls and boys by maternal and paternal classification, as well as actual weight status; Health study, Germany

Figure 1 shows the frequency of children’s weight categorisation according subjective maternal classification, subjective paternal classification and actual weight status according to German reference values [31]; Health study, Germany.

**Fig. 2:**
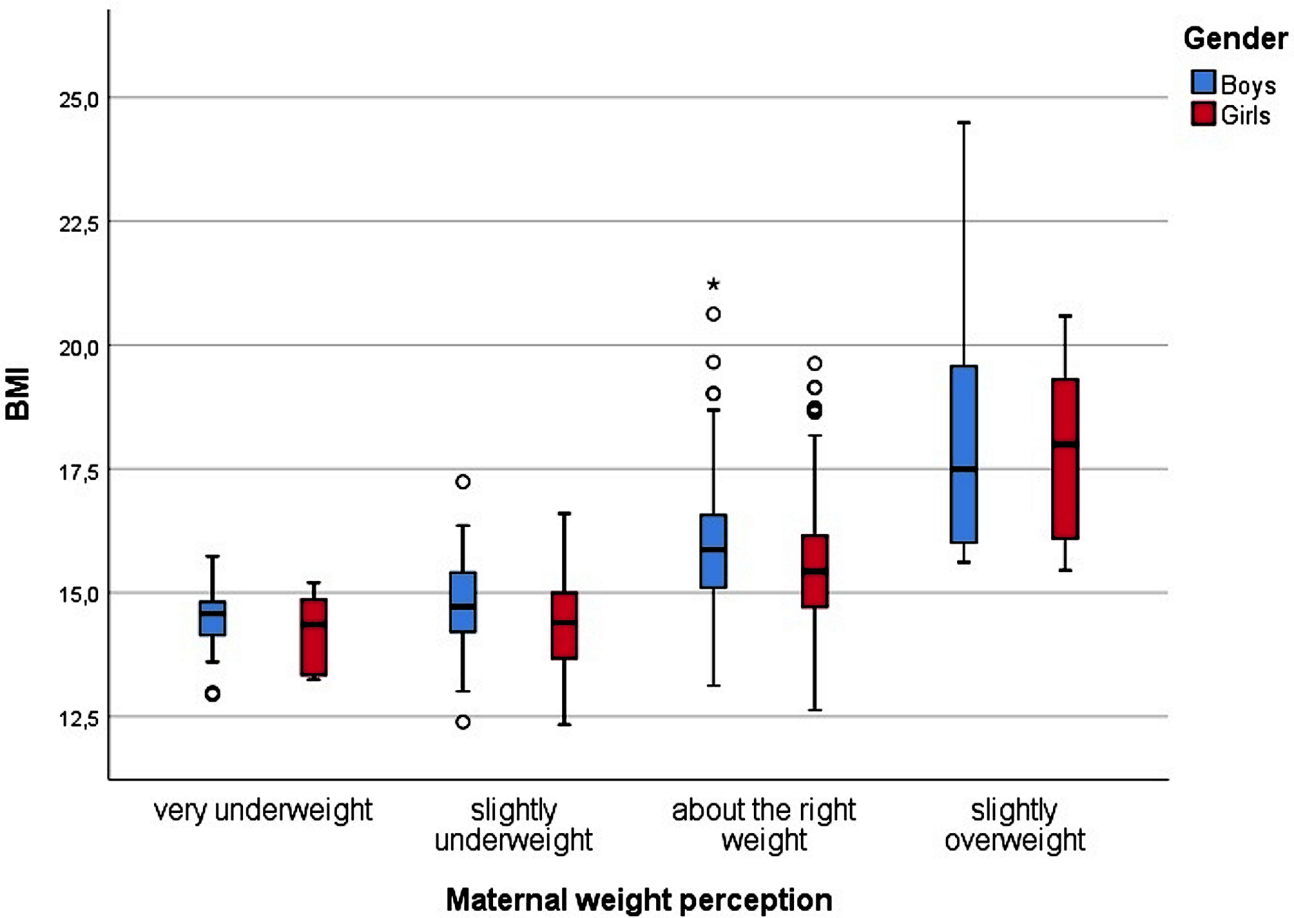
Distribution of measured BMI for girls and boys by maternal classification of child weight status; Health study, Germany

**Fig. 3:**
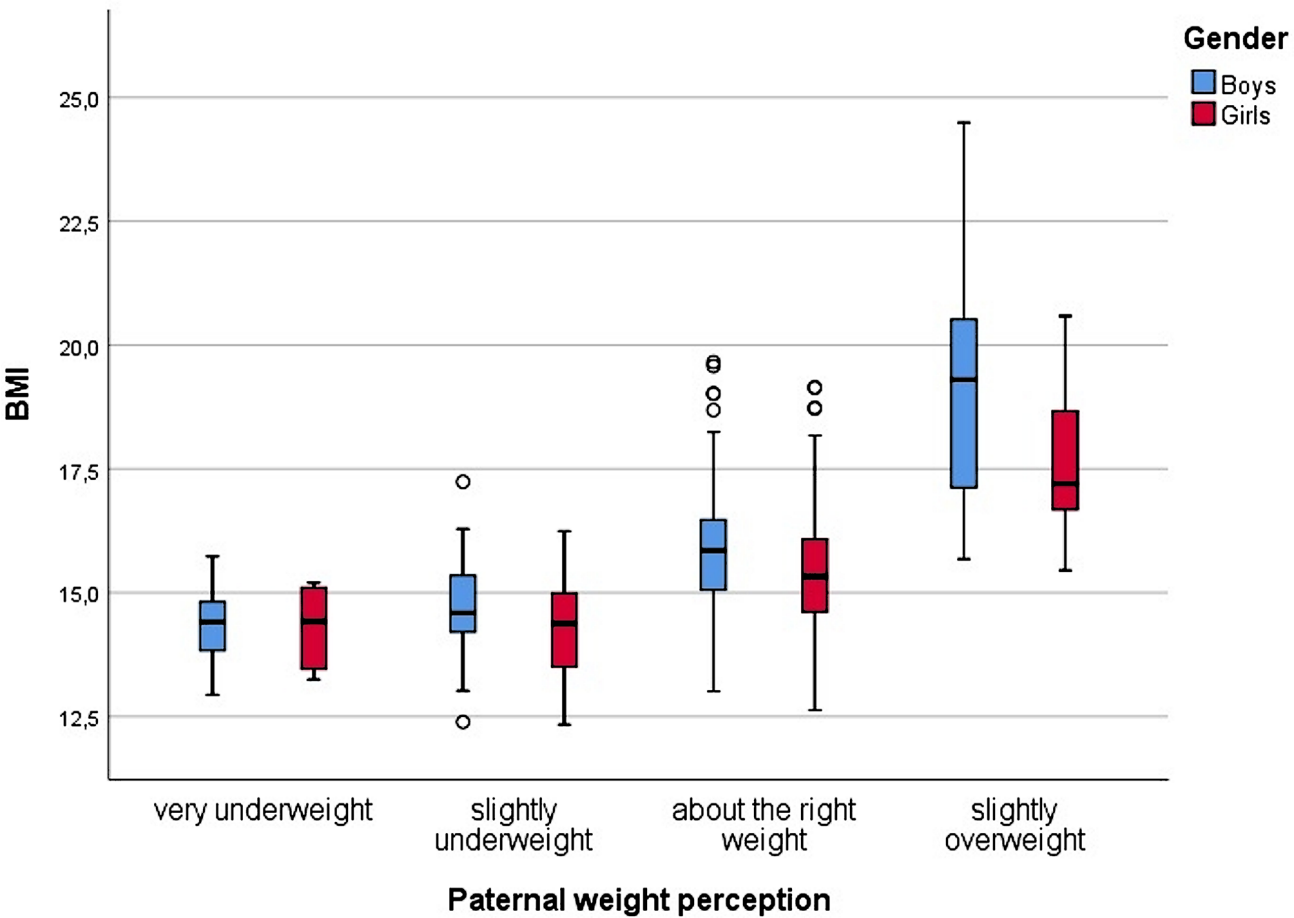
Distribution of measured BMI for girls and boys by paternal classification of child weight status; Health study, Germany

Figures 2 and 3 present vertical Tukey boxplots [38] of boys’ and girls’ BMI by maternal and paternal classification category (which can be seen in Fig. 1) an shows an overlap in distribution of objectively measured child BMI and subjective maternal and paternal classification. Mean BMI is slightly higher for boys than for girls (15.68 vs. 15.45, *p* = 0.005).

Kendall’s tau as well as Spearman’s rho showed significant correlation between childhood weight (BMI percentiles) and maternal (τ = 0.386, *r*_s_=0.457) and paternal (τ = 0.355, *r*_s_=0.439) classification (all *p* < 0.01). Effect sizes are *R*_s_²=0.209 for maternal and *R*_s_²=0.193 for paternal perception. The Wilcoxon test statistic is *z*=-19,924 for maternal and *z*=-20.040 for paternal perception. The central tendencies of the two measurement times differ significantly (asymptotic Wilcoxon test: *Z*=-3.75, *p* < 0.001), which shows that parental perception of their child’s weight status and the child’s actual weight differ significantly from another.

Objective BMI measure and subjective parental classification of child’s weight status were compared for each individual in the sample to assess the extent of parental misclassification. Concerning mothers, 30.1% of them had a false perception (26.5% false negative, 3.6% false positive). Regarding fathers, 27.0% had a false perception (22.6% false negative, 4.4% false positive). Further analysis concentrated on *false negative* perception, which represents the case that the child is objectively in a higher weight category (according to German reference values [39]) than subjectively classified by their parent.

In the following, *false positive* perceptions build one category together with *right* (meaning consistent) perceptions. In an attempt to uncover potential reasons for these high percentages of false negative perceptions, a range of possible determinants are considered in the regression analysis. As the main determinants of interest, the level of education, the parental obesity status, the presence of migration background and family income were defined as covariates (prevalences see Fig. 1).

When analysed separately, paternal as well as maternal overweight was significantly related to false negative classification (paternal: OR: 1.522, CI: 1.014–2.283; p < 0.05; maternal: OR: 1.841, CI: 1.276–2.656; p < 0.001). Yet, in the binary logistic regression, only the misclassification of the partner and the weight category of the child were predictors for parental *false negative* classification of children’s weight status (see Table 3). The test for linearity of the logit showed linearity of weight category of the child (with a mean of 1.61 and a standard deviation of 1.92, with no gender difference), so the higher the child’s weight status, the more likely it is for the parents to perceive their child’s weight status incorrectly.

**Table 3:** Binary logistic regression of false negative classification of child weight status by parents; Health study, Germany

Predictors	Maternal false negative classification (n = 657)			Paternal false negative classification (n = 614)		
	OR	95% CI	*p*	OR	95% CI	*p*
Misclassification by the partner	184.51	85.08; 400.11	0.001	184.51	85.08; 400.11	0.001
Child is overweight and/or obese	5.86	1.06; 32.27	0.042	5.35	1.12; 25.57	0.036

*OR* odds ratio, 95% CI *95% confidential interval*

## Discussion

In this study, 10% of kindergarten children were overweight (including 2% obese children), whereas only 2.6% and 2.7% of mothers and fathers, respectively, classified their child as “slightly overweight” and none as “very overweight”. Overall, 26.5% of mothers and 22.6% of fathers had a false negative perception of their child’s weight status. This goes in line with a systematic review that showed that 86% of parents of 2–6 year old children underestimate their child’s excess weight [40]. Yet, it is important for parents to recognise their children’s excess weight since it has been suggested that parental inability to recognise weight change and overweight in children may contribute to an increase in childhood obesity levels [41] as no necessity for behaviour change is seen.

The WHO has set the global target to halt the rise in obesity by 2025 [2]. So far, national policy actions to prevent obesity and to drive systemic changes have mostly failed [2]. The pandemic of obesity has recently been reframed as part of *the global syndemic*: the co-occurring, interacting pandemics of obesity, undernutrition, and climate change that are driven by common underlying societal drivers [42]. Addressing childhood obesity in an equitable manner requires a comprehensive approach that takes into consideration its complex causes.

Parents can play an important role in supporting healthy childhood development, including their weight management [34, 35]. However, studies have found that parents may not be able to accurately perceive their children’s weight status [43–46].

A recent review by the WHO [47] reported, that 82.3% and 93.8% of parents underestimated their child’s weight status in the overweight (excluding obesity) and obesity categories, respectively. They also reported significant differences in misclassification driven by family education level, whereby low to medium education resulted in an underestimation of boys’ more than of girls’ weight status [47]. This cannot be confirmed by these results as family education was significantly related to parental as well as child overweight but not to parental misclassification of their child’s weight status. However, it is known, that social disadvantage has an impact not only on children’s weight status but also on other aspects of their health development. Previous studies indicate, for example, an increased incidence of early childhood developmental delays and health disorders as well as accidental injuries and dental problems among children from families with a socially disadvantaged background [48]. Also behaviourally correlated risk factors such as smoking, lack of physical activity and obesity, which are key for the majority of deceases and deaths occur in middle and older age, accumulate in those families (parents and children) with a low socio-economic background [49]. This is even more worrying considering that in Germany, the poverty rate continues rising, whereas in most other welfare states a much lower increase or even a decrease is observed [50].

Further, in this study, it showed to be more likely that overweight parents underestimated their children’s excess weight, in the robust analyses however, only misclassification of the partner and the child’s weight status were significant predictors for parental false negative classification of children’s weight status.

Various factors seem to affect parents’ perceptions of their child’s weight status including their age, parental weight status, and population prevalence of obesity [40]. Yet, results are inconsistent, especially when considering parental weight status. These results show when analysed separately, a strong association between parental weight status and false negative classification of their child’s weight status, suggesting that parents who are overweight themselves misclassify their child’s weight status more often false negatively. A Chinese study [51] on the other hand, showed opposite findings with low maternal weight being associated with maternal underestimation of child weight status. The authors suggested as an explanation that potentially, mothers, who are overweight themselves, may have a better understanding of what being overweight means. However, they also found out that the more overweight a child was, the greater the odds that their mothers would underestimate their overweight status [51]. This goes in line with findings of this study, where results suggest that the higher the child’s weight status, the more likely it is for their parents to have an incorrect perception. Similar findings have been reported for five-year old preschool children, where parents accurately identified over 90% of underweight children, but tended to underestimate normal weight and overweight children’s weight status [52]. Since it has been shown that population prevalence of obesity is also associated to parents’ (mis)perception of their child’s weight status [53], it may reflect perceived “normality” or reluctance to recognize or admit that their child is overweight. This however, needs to be investigated further.

Parents play an important role in regulating their children’s health behaviours by providing a healthy diet or encourage their children to be sufficiently physically active. Nearly a third of obese preschool children, and approximately half of obese school-age children, become obese adults [54], which imposes high costs on the health care system [55]. However, in order to take their child to see a doctor about their potentially unhealthy weight, the parent has to recognise that their child is overweight or obese. Parental misclassification occurs if parents of objectively overweight children perceive their child as normal weight. Therefore, they may not modify the child’s diet, promote their child’s physical activity, or seek or even disregard medical advice. Consequently, childhood obesity has to be identified in order to be treated.

Nevertheless, although this study has a large sample size, which increases the odds of having sufficient power to detect differences, this study is not without limitations, which should be considered when interpreting these results. This study faces potential sources of bias, such as a non-response bias but also that parental height and weight was based on self-report, which might have led to downward bias and misclassification of weight status. Nevertheless, children’s anthropometric data was objectively measured in this sample by trained stuff according to the ISAK-standards [31, 33], which makes up a notable strength in data acquisition. Parental data, even if subjective, were analysed for both, mother and father and considering the large sample size and high response rate, it can be assumed that the sample is representative for the state of Baden-Württemberg and southwest Germany. Still, even is robust analyses were chosen, some results show very large odds ratios or standard deviations, which should therefore be interpreted carefully.

To our knowledge, there are no other studies on childhood overweight using a syndemic approach, yet. However, this framework is perfectly suitable to identify interactions, especially to break down potential inequalities. The few existing studies focus on interactions between urban environment and neighbourhood safety on childhood overweight [56, 57], which is undoubtedly an important research area. Still, since children – especially at a young age, such as in this study – are primarily socialised in their families, who are responsible for providing nutrition and support for physical activity and other health behaviours, it is important to increase parents’ knowledge on what constitutes healthy weight, as well as the potential harm of overweight for children and their development. Therefore, further research is needed in order to identify the truly underlying factors of health inequality to enable parents and families to gain knowledge and especially competencies to provide children with a healthy upbringing, independent of their social and familial background. Up to then, children and young people who grow up in unfavourable living conditions should be an important target group for prevention and health promotion, and such measures have to start very early and should be low-threshold so everyone is reached and understood.

## Conclusion

Children from parents with low educational background have an elevated risk of overweight, already at a very young age. Education in general (not explicitly health education) seems to play a tremendous role in the prevention of overweight and obesity and should therefore be implied in policies enhancing health equity. It is known, that universal programmes can be powerful, but to tackle inequities successfully, accelerated programmes are needed, including support during pregnancy and the early years of life [46]. Training healthcare providers and teachers in kindergartens to work with parents to recognize unhealthy weight in children would be valuable. This study adds support to a theory of syndemics among the field of obesity research, which suggests that synergistically related biological, psychological, social, and behavioural factors disproportionately affect health and health-related behaviours.

## Data Availability

The datasets used and analysed during the current study are available from the corresponding author on reasonable request.
